# Biocidal Activity of Metal Nanoparticles Synthesized by *Fusarium solani* against Multidrug-Resistant Bacteria and Mycotoxigenic Fungi

**DOI:** 10.4014/jmb.1906.06070

**Published:** 2019-08-27

**Authors:** Manal T. El Sayed, Ashraf S.A. El-Sayed

**Affiliations:** Botany and Microbiology Department, Faculty of Science, Zagazig University, 44519, Egypt

**Keywords:** Antimicrobial activity, characterization, *Fusarium solani*, nanoparticles, *Pseudomonas aeruginosa*, pyocyanin

## Abstract

Antibiotic resistance by pathogenic bacteria and fungi is one of the most serious global public health problems in the 21^st^ century, directly affecting human health and lifestyle. *Pseudomonas aeruginosa* and *Staphylococcus aureus* with strong resistance to the common antibiotics have been isolated from Intensive Care Unit patients at Zagazig Hospital. Thus, in this study we assessed the biocidal activity of nanoparticles of silver, copper and zinc synthesized by *Fusarium solani* KJ 623702 against these multidrug resistant-bacteria. The synthesized Metal Nano-particles (MNPs) were characterized by UV-Vis spectroscopy, transmission electron microscopy, Fourier transform infrared spectroscopy, X-ray diffraction, and Zeta potential. The Fourier transform infrared spectroscopy (FTIR) result showed the presence of different functional groups such as carboxyl, amino and thiol, ester and peptide bonds in addition to glycosidic bonds that might stabilize the dispersity of MNPs from aggregation. The antimicrobial potential of MNPs by *F. solani* against the multidrug-resistant (MDR) *P. aeruginosa* and *S. aureus* in addition to the mycotoxigenic *Aspergillus awamori*, *A. fumigatus* and *F. oxysporum* was investigated, based on the visual growth by diameter of inhibition zone. Among the synthesized MNPs, the spherical AgNPs (13.70 nm) displayed significant effect against *P. aeruginosa* (Zone of Inhibition 22.4 mm and Minimum Inhibitory Concentration 21.33 μg/ml), while ZINC oxide Nano-Particles were the most effective against *F. oxysporum* (ZOI, 18.5 mm and MIC 24.7 μg/ml). Transmission Electron Microscope micrographs of AgNP-treated *P. aeruginosa* showed cracks and pits in the cell wall, with internalization of NPs. Production of pyocyanin pigment was significantly inhibited by AgNPs in a concentration-dependent manner, and at 5-20 μg of AgNPs/ml, the pigment production was reduced by about 15- 100%, respectively.

## Introduction

Antimicrobial resistance (AMR) is a major current global health threat, estimated to be responsible for over 700,000 deaths annually [1]. It is expected that nearly 10 million people may die every year by 2050 due to multidrug-resistant (MDR) infection [2]. Significant economic losses correlated with the impact of mycotoxins on human health, animal productivity, and both native and international commerce (FAO 2001) have been reported. Exploration and development of new antimicrobial strategies constitute a crucial challenge in controlling the spread of AMR (WHO 2018). The oligo dynamic effect of silver nanoparticles (AgNPs) could be essential in the development of MDR bacteria-regulating medications, replacing other mainstream therapeutics [3]. Biosynthesized AgNPs have antibacterial potential against the growth of MDR *Staphylococcus aureus*, *Salmonella typhi*, *Streptococcus pyogenes*, *Pseudomonas aeruginosa* and *Escherichia coli* [4, 5]. The antimicrobial properties of metal NPs could be mainly attributed to the following: 1) Interaction with the plasma membrane and inhibition ATPase activity, decreasing the cellular ATP and distorting cellular respiration and permeability, 2) damage to DNA backbone, preventing its replication by denaturing the ribosomes, 3) generation of Reactive Oxygen Species through interaction with biomolecules and/or enzymes leading to cell destruction, and 4) change of microbial signal transduction pathways [6-8]. Zinc oxide nanoparticles (ZnONPs) have a strong antifungal activity against *Aspergillus* and *Penicillium* (Shobha *et al*. 2019), while copper nano-particles (CuNPs) have displayed strong activity against MDR bacteria such as *E. coli*, *S. aureus*, and *Candida albicans*. The adsorption of CuNPs by the microbial cell wall leads to the formation of a thin Cu oxide layer reducing the ability of microorganisms to develop resistance against them [9]. Several physical and chemical approaches have been applied in the synthesis of NPs [10-12]. However, these methods usually need high temperature and a vacuum, which usually makes them incompatible with a sustainable ecosystem due to the generation of toxic byproducts [13]. Therefore, simple, green, rapid and effective biological approaches have been chosen for the synthesis of NPs. These NPs are economically feasible, non-toxic, environmentally friendly and have higher biological compatibility with well-defined size and shape under optimized conditions [14, 15]. Moreover, the higher stability and solubility of biogenic NPs in water are among the advantages of biological methods [7]. There are three main constituents involved in NP biological synthesis: a metal precursor, the reducing agent and a stabilizing/capping agent that is nontoxic [8]. Biomolecules such as vitamins, proteins, sugars, phenolic compounds, poly-saccharides and others are believed to be responsible for the reduction of charged metallic ions to their zero-valent nano-forms, effectively wrapping NPs to prevent their agglomeration [16]. Mechanisms of NP biosynthesis including the activity of nitrate reductase, electron shuttle quinones and the combination of both are reported for the myco-mediated synthesis of NPs [17]. Moreover, the participation of NADH/NADPH-dependent reductase in the metal bioreduction process has also been reported [18]. Wanarska and Maliszewsk [18] suggested that polypeptides are the main molecules involved in biomineralization of Ag^+^ to AgNPs by *P. cyclopium*. Aromatic amino acids, such as tryptophan and tyrosine play a great role in synthesis of metallic NPs through their amino and/or carboxylate groups [19].

The biosynthesis of metal NPs by fungi does not require much downstream processing and appears to be an easy and cost-effective approach [20], having a higher affinity towards a broad range of heavy metals [21]. This higher fungal potency in the synthesis of NPs is due to their higher yields of extracellular enzymes, proteins and aromatic compounds (naphthoquinone and anthraquinone) which act as an electron shuttle in metal ion reduction [22]. The hydroxyl and carboxyl groups in tyrosine and asparagine and/or glutamic residues are demonstrated to be implemented in synthesis of AgNPs [23]. The potentiality for synthesis of metal NPs by different fungal genera has been extensively reported [24, 25]. Although few studies reporting the biosynthetic potency of metal NPs by *Fusarium solani* are documented, the fast growth of this fungus on soils heavily contaminated with heavy metals and industrial pollutants is recognized. Thus, in this study we assessed the potentiality of *F. solani* for reduction of various metals and synthesis of their corresponding nanoparticles, especially AgNPs, CuNPs and ZnONPs. In addition, we evaluated the biological activity of these metal NPs by *F. solani* against various MDR bacteria and mycotoxigenic fungi.

## Materials and Methods

### Fungal Strain, Culture Conditions and Synthesis of Metal NPs

*F. solani* KJ 623702 was previously isolated from soil receiving the long-term application of industrial effluents as irrigates and identified according to morphological characteristics and its rDNA sequence (18S-28S rRNA, flanking ITS 1, 5.8S rRNA, and ITS 2) [26]. The sequence of *F. solani* has been submitted to GenBank with accession number KJ623702. To prepare the biomass for NP synthesis, the fungal strain was cultured at 25°C for 5 d and 120 rpm in the PDB (potato dextrose broth) containing (g/1): potato extract from 200 g, dextrose 20 g and pH 5.1 ± 0.2. The fungal mycelium was harvested from the potato dextrose broth by centrifugation (5,000 ×*g*, 20 min and 4°C) and washed three times with deionized water to remove any media components.

For metal NP synthesis, 10 g of fresh biomass was suspended in 100 ml of sterilized deionized water, incubated for 48 h at 28°C under shaking (120 rpm), and the mycelia were collected by filtration and centrifugation to obtain the cell-free filtrate (CFF). Fifty ml of the CFF was mixed separately with 50 ml of freshly prepared 1 mM AgNO_3_, 1 mM CuSO_4_, and 0.01 mM ZnSO_4_ as a final concentration, and then incubated for 24 h at 28°C under shaking at 120 rpm in the dark. The development of AgNPs and CuNPs was assessed from the visual inspection of the intensity of yellow to brown color and green-blue color of the reaction solution, respectively. The white precipitate due to ZnONPs formation was observed. The NPs were collected by centrifugation, re-dispersed in sterilized deionized water, air-dried to a definite weight, resuspended in sterilized deionized water and stored at 4°C in dark till use.

### Characterization of Metal NPs

The reduction of metal ions was assessed by T80 UV-Vis spectrophotometer at a resolution of 1 nm from 200 normalizing to controls. The zeta potential of NPs was determined in the range of -200:200 mV by Zetasizer Nano series (UK) at Nanotechnology Centre, Agriculture Research Centre, Giza, Egypt. Negative control of metal precursors dissolved in sterile distilled water was used. The morphology and size of the synthesized NPs were investigated using a transmission electron microscope (TEM) (JEOL-1010 electron microscope, Japan) at the Regional Center of Mycology and Biotechnology, Cairo, Egypt, operated at an accelerating voltage of 100 kV. Ten microliters of NP solution were dropped on a carbon-coated copper grid and allowed to dry at room temperature.

### X-Ray Diffraction (XRD) Measurements

The crystal structures of the synthesized NPs were analyzed on a drop-coated glass substrate and recorded on a Broker D8 advanced target Cukα powder diffractometer (λ = 1.5418Å) over the range 0-80o 2θ (Central Metallurgical & Development Institute, Helwan, Egypt) for confirmation of the crystalline nature. The crystallinity index, Icry of NPs was determined [27] according to the following equation:

$$I_{\mathrm{cry}}=D_{p}(\mathrm{TEM},\mathrm{SEM})/D_{\mathrm{cry}}(\mathrm{XRD})(I_{\mathrm{cry}}\geq 1.0)$$

where D_p_ is the particle size obtained from either SEM or TEM morphological analysis, D_cry_ is the particle size determined from the XRD. If I_cry_ is close to 1.0, then it is assumed that the crystallite size represents monocrystalline, while polycrystalline has a larger crystallinity index [28].

### Fourier Transform Infrared (FTIR) Spectroscopy

FTIR spectra of the NPs were performed to assess the possible functional groups involved in stabilization of NPs. The freezedried NPs were examined in KBr (as a binding agent) in the range of 400-4,000 cm^-1^ with a PerkinElmer FTIR 1650 spectrophotometer (Center of Microanalysis, Cairo University, Egypt).

### Antimicrobial Activity of Synthesized NPs

Bacterial isolates were obtained from different medical specimens from the wounds of patients admitted to Zagazig University Hospital, Zagazig, Egypt, during the period from January to July 2018. Under aseptic conditions, the specimens were processed by the Bacteriology Lab, Botany and Microbiology Department, Faculty of Science, Zagazig University. The grown colonies were identified based on their morphological and biochemical tests according to Bergey’s manual [29-31]. For detecting the antibacterial resistance, nineteen antibiotics “ceftazidime, cephalexin, azithromycin, doxycycline, penicillin, amoxicillin, vancomycin, amikacin, aztreonam, cefotaxime, Imipenem, ciprofloxacin, chloramphenicol, nitrofurantoin, oxacillin, erythromycin, gentamicin, trimethoprim/sulphamethoxazole and amoxicillin/clavulanic acid were selected. The antibacterial activity of the synthesized NPs was performed by the diskdiffusion method (Bauer *et al*. 1966) following the CLSI guidelines.

The mycotoxigenic fungal isolates *Aspergillus awamori* JQ695830.1, *A. fumigatus* JX006238 and *Fusarium oxysporum* FR11 used in the antimicrobial assay were obtained from Enzymology and Fungal Biotechnology Lab, Botany and Microbiology Department, Faculty of Science, Zagazig University.

The antifungal and antibacterial activities of the synthesized NPs were assessed by the disk-diffusion method [32], following the CLSI guidelines. Itraconazole disc (10 μg), ampicillin disc (10 μg) and ciprofloxacin disc (5 μg) were used as positive controls for fungi, gram-positive bacteria and gram-negative bacteria, respectively. Two bacterial isolates showed the highest resistance to three or more antimicrobial categories (MDR) grown on nutrient broth (24 h at 37°C) to prepare cell suspensions of 10^8^ CFU/ml. The fungal strains were cultured on potato dextrose agar slants at 28°C for five days. Spores were harvested by adding 10 ml of sterile distilled water containing 0.05% Tween 20 and scraping the surface of the culture to free the spores. The spore suspensions were adjusted with sterile 0.05% Tween 20 to give a final concentration of 10^5^ conidia/ml.

To determine the zone of inhibition (ZOI), one ml of bacterial cell suspensions and fungal spore suspensions were seeded independently into Mueller–Hinton agar (MHA) and PDA media, respectively, shaken vigorously and then poured. After medium solidification, sterilized Whatman’s filter paper discs (6 mm diameter) impregnated each with 20 μl of the different concentrations of AgNPs, CuNPs and ZnONPs placed on the surface of seeded plates. Twenty μl of *F. solani* CFF was used as a negative control, while the antibiotic discs were used as a positive control. ZOI was measured in mm. All the experiments were performed in triplicates, with the results expressed as mean ± SD.

To estimate the minimum inhibitory concentration (MIC), 10 μl of the bacterial suspension was added individually to 1 ml of nutrient broth. Different concentrations of NPs (5, 10, 15, 20, 25, 30, 35, 40, 45, and 50 μg/ml) were added and incubated 37°C for 24 h. Fifty ml of PDB was inoculated with 200 μl of fungal spore suspension at 28°C for seven days. The MIC values correspond to the concentrations that inhibit 99% of the microbial growth [33].

### TEM Investigation

*P. aeruginosa* pellets treated with the sub-MIC dose of AgNPs were harvested by centrifugation (6,000 ×*g* at 4°C for 15 min) and washed with distilled water thrice. Then they were prepared for TEM investigation and examined by a JEOL-1010 electron microscope (Regional Center of Mycology and Biotechnology, Egypt).

### Pyocyanin Assay

Different concentrations of AgNPs (5, 10, 15, and 20 μg /ml) were added to 250-ml Erlenmeyer flasks containing nutrient broth. The flasks were autoclaved for 20 min at 121°C, cooled at room temperature and inoculated with the bacterial suspension of *P. aeruginosa*, then incubated at 37°C for four days. Pyocyanin was then extracted from culture filtrates of untreated and AgNP-treated *P. aeruginosa* and measured by the method of [34]. Three ml of chloroform was added to 5 ml of culture supernatant and mixed. Then the chloroform layer was mixed with 1 ml of 0.2 M HCl. After centrifugation, the top layer (0.2 M HCl) was removed. Pyocyanin was quantitatively assayed based on measuring the absorbance at 520 nm [35] according to the following equation:

Pyocyaninconcentration(μg/ml)=O.Dat520×17.072

### Statistical Analysis

All data were statistically analyzed applying the General Linear Model procedure of the SPSS ver. 18 (IBM Corp., USA). The significance of the difference between treatment groups was determined by Waller-Duncan k-ratio. All statements of significance were based on the probability of *p* < 0.05. The ANOVA test was carried out in the BioEstat 5.3 program [36].

## Results and Discussion

### Characterization of AgNPs, CuNPs, and ZnONPs

**Fig. 1:**
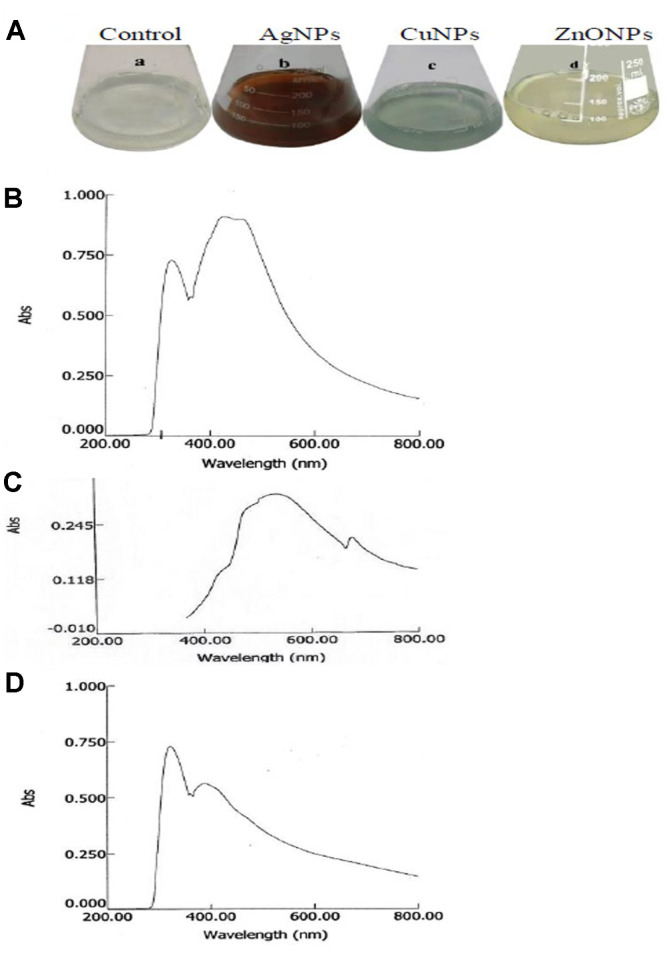
Biosynthesis of metal nanoparticles by *F. solani*. The fungus was grown for 6 days, the mycelial pellets were collected, washed with distilled water for two hours then filtered. The washedoff water was amended with 1 mM AgNO_3_, CuSO_4_, and ZnSO_4_, then the visual color was photographed after 10 h (**A**), UV-Visible spectra of AgNPs (**B**), CuNPs (**C**), and ZnONPs (**D**).

The biosynthetic potency of AgNPs, CuNPs, and ZnONPs was detected from the visible coloration of the reaction mixture (CFF+ metal ion precursor). The dark brown color, green-blue color, and coalescing white suggested the formation of AgNPs, CuNPs, and ZnONPs, respectively. The color change was due to the excitation of surface plasmon vibrations resonance (SPR) with NPs in the visible region [37]. The positive and negative controls maintained their original colors which gave insight into the fact that the formation of NPs requires both CFF and metal precursors. The CFF contained enzymes and proteins. The enzymes reduced the metal ions into metal atoms, while the proteins (Fig. S1) acted as capping agents for stabilizing the metal atoms [38]. The lack of precipitation or agglomeration ensured the stability of NPs due to the presence of capping agents that might be sugars or proteins [39]. UV-Visible spectra of AgNPs, CuNPs, and ZnONPs showed peaks at 422 nm, 675 nm, and 375 nm (Fig. 1), respectively, consistent with those reported by [40, 7, 8]. The area and localization of λ_max_ of SPR depend on the shape, particle size, aggregation state, precursor concentration, reaction temperature, type of solvent, and surrounding dielectric medium [41].

**Fig. 2:**
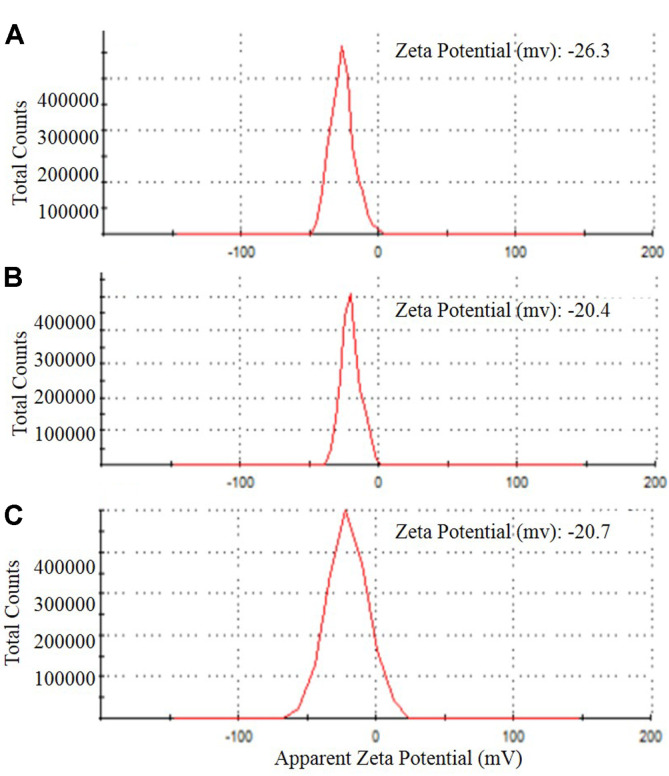
Z- potential values of synthesized metal NPs; AgNPs (**A**), CuNPs (**B**), and ZnONPs (**C**) synthesized by *F. solani*.

**Fig. 3:**
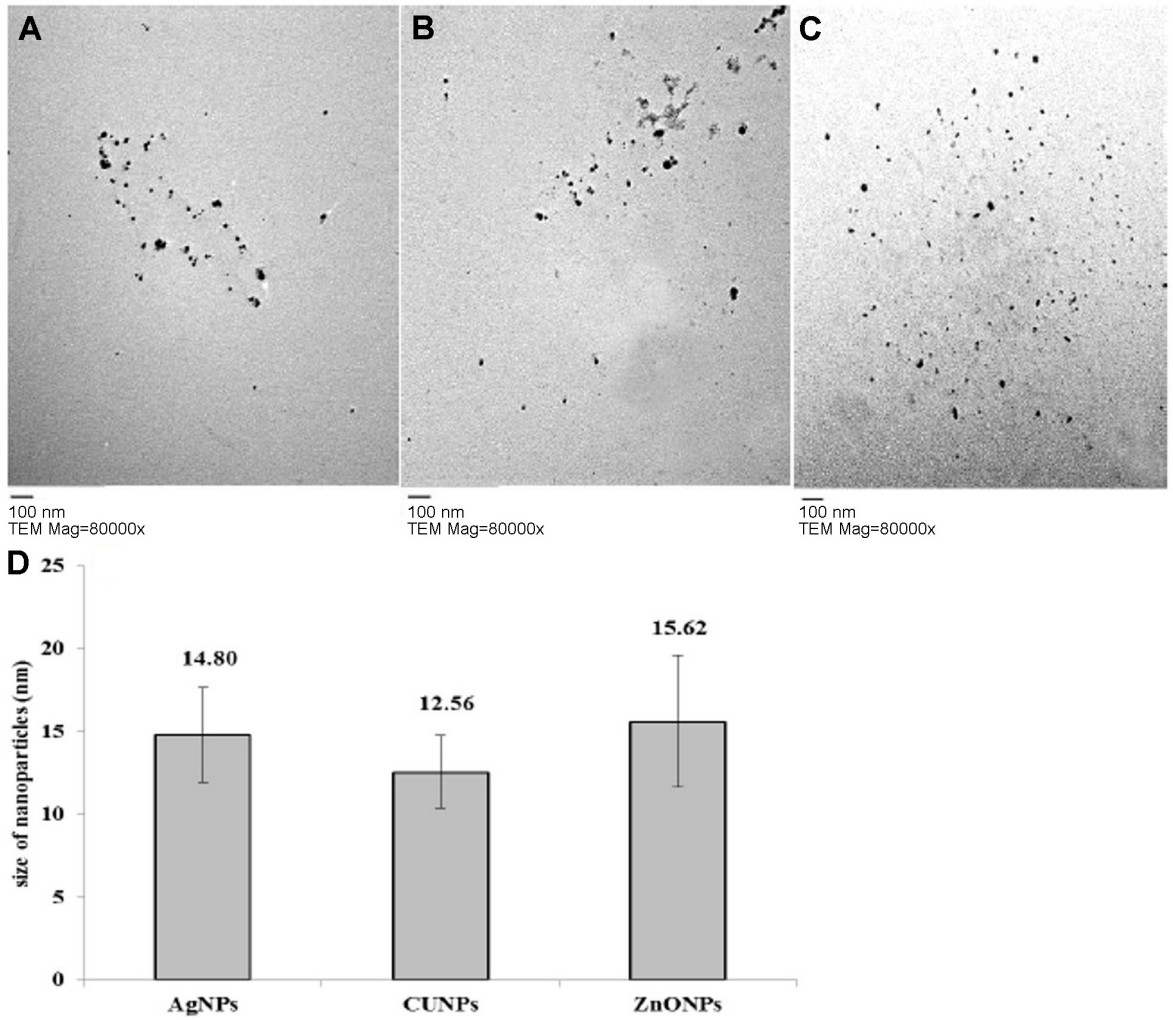
The scale bar is 100 nm, TEM Mag = 80,000× of synthesized metal NPs; AgNPs (**A**), CuNPs (**B**), and ZnONPs (**C**) and overall molecular sizes (**D**) synthesized by *F. solani*.

The surface charge potential, or Zeta potential, plays a crucial role in the stability of NPs in aqueous solution and is defined as the difference in potential between the dispersing medium and the stationary layer of fluid attached to the dispersed particle. In the present study, Z-potential values of AgNPs, CuNPs and ZnONPs were -30.9, -34.8, and -25.3 mV, respectively, indicating that the biogenic NPs were moderately stable at room temperature (Fig. 2). Zeta potential is an indicator of the degree of repulsion/attraction between NPs [42]. The size and shape of NPs greatly influence their antimicrobial effect [43]. The diameters of AgNPs, CuNPs, and ZnONPs ranged from 7.65 to 18.89 nm (13.70 nm average size), 9.97 to 19.49 nm (13.42 nm average size), and 8.55 to 21.76 nm (17.33 nm average size), respectively, and they were spherically shaped (Fig. 3). The edges of mycosynthesized NPs were lighter than the centers, suggesting that biomolecules such as proteins capped the NPs [44]. The difference in particle size may be due to the formation of NPs at different times [45].

### X-Ray Diffraction (XRD)

**Fig. 4:**
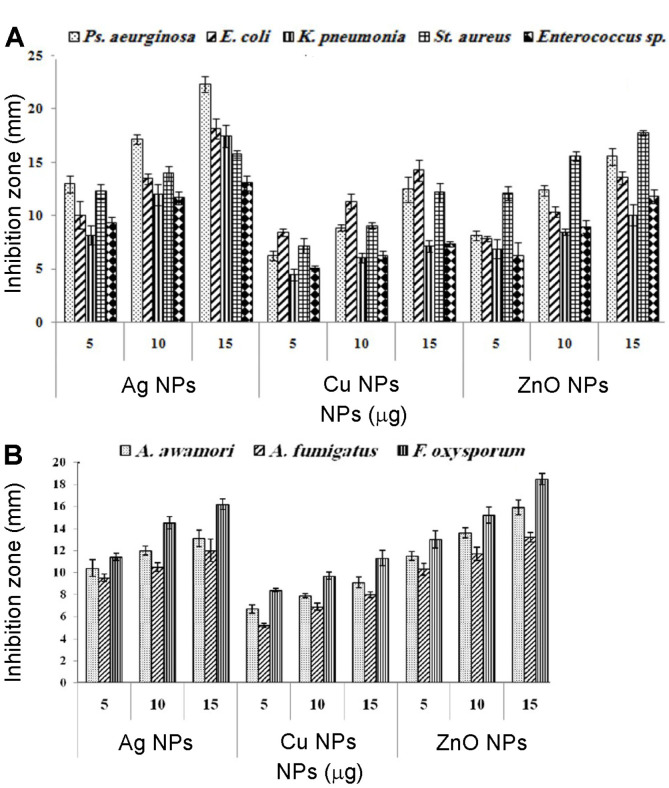
Antimicrobial activity of *F. solani* synthesized AgNPs, CuNPs, and ZnONPs (5, 10, and 15 μg) against various multidrug resistant bacteria (**A**) and mycotoxigenic fungi (**B**) as revealed from the diameter of Zone of inhibition (ZOI).

The XRD pattern of AgNPs showed eleven peaks distributed from 27.3 to 54.99° of 2θ. There are three intense peaks at 27.3°, 29.30°, and 33.29° of 2θ indicating that (125),(226), and (264) sets of lattice planes, respectively, were present. The average crystal size of AgNPs was 18.26 nm. Four intense peaks at 30.73°, 28.25°, 33.13°, and 35.79° of 2θ are present (Fig. S2). They belong to (110), (-111) and (111) planes of Cu_2_O, respectively. There are less intense peaks at 2θ 37.3°, 40.20°, and 43.24° of 2θ which belong to (111) planes of CuO. The average particle size of CuNPs was 3 nm. The XRD pattern of ZnONPs (Fig. 4C) showed seven intense peaks at 31.60°, 45.41°, 28.30°, 30.20°, 40.41°, 56.40°, and 75.19° of 2θ indicating that (100), (101), (111), (102), and (112) sets of lattice planes, respectively, are present. The average particle size of ZnONPs was 51.34 nm. CuNPs were polycrystalline with I_cry_ > 1, while AgNPs and ZnONPs were monocrystalline with I_cry_ < 1.

### FTIR Spectroscopy

FTIR analysis was carried out to clarify the possible interactions between metal ions and bioactive molecules. The FTIR spectra of the native CFF of *F. solani* KJ 623702 and the synthesized AgNPs, CuNPs, and ZnONPs were demonstrated (Fig. S3). The shift at 3428.81 cm^-1^ in the case of CuNPs indicated the role of N-H/C-H/O-H stretching of amines and amides I and II in the synthesized metal NPs [46, 47]. Changes at 2,928.38 and 1,384.64 cm^-1^ and appearance of new peaks at 2,857.99, 2,403.83, and 1,545.67 cm^-1^ (AgNPs) were assigned to the C-H stretching vibrations of protein methylene groups, O-H stretching of carboxylic acids and

N-H bending. A shift at 2,371.05 cm^-1^ was indicative of the role of nitrogen compounds (showing triple or cumulative double bonds such as nitriles and cyanates) and sulfur compounds (like amino acids). A significant shift at 1,646.91 cm^-1^, particularly in CuNPs and ZnONPs, is representative of protein and indicated the involvement of C=O and N-H bending for amides I and II in NP synthesis [49]. New peaks at 1,432.85 cm^-1^ (CuNPs) and 1,454.10 cm^-1^ (ZnONPs) revealed that alkanes and –CH2/CH3 bending vibrations in lipids and proteins, respectively, are involved in the process. The disappearance of the peak at 1,384.64 cm^-1^ (ZnONPs) attributed to the –H–N–C=O stretching vibration of the amide III bands of the protein [50]. Wen *et al*. [51] demonstrated that the peaks at 3,433, 1,637, and 1,383 cm^-1^ confirmed that amides are present on the surface of NPs. The amide bonds between amino acid residues in proteins result in the well-known signatures in the infrared region of the electromagnetic spectrum [52]. Shifts at 1,239.04 and 1,081.87 cm^-1^ (especially in the case of ZnONPs) and the new peak at 1,138.12 cm^-1^ (CuNPs) were specified to C-O, alkyl amine, alkyl ketone and C-O-C of polysaccharides [53]. Furthermore, the new peak at 981.59 cm^-1^ was due to -CH=CH, C-Cl. The new bands at the wave number 830.21 (AgNPs), 713.53 cm^-1^ (CuNPs), and 800.30 cm^-1^ (ZnONPs) represented the fingerprint region for the α-glycosidic bond in carbohydrate and N–H bending vibration, respectively [54, 55]. From these observations, we concluded that glycoprotein containing polysaccharide with α-glycosidic bond and protein likely capped the NPs. Similar studies indicated that linkages like –C–O–C–and C–N or functional groups such as amide derived from heterocyclic compounds such as amino acids were present in the CFF of fungi, acting as the capping ligands and adsorbed strongly to the NPs [56, 57]. The disappearance of the peak at 875 cm^-1^ strongly indicated the intervention of phosphorous and P=S stretching in AgNP synthesis [58]. The new peaks around 600, 413, 428, 450, and 474 cm^-1^ pointed to the involvement of metal-O stretching vibration. The FTIR peaks revealed that sulfur, nitrogen, and phosphorus-containing compounds, glycoprotein-containing polysaccharides with α-glycosidic bond, and protein with β-sheet and a carbonyl group of amino acid residues were involved in the synthesis of biogenic NPs.

### Isolation of Multidrug-Resistant Bacteria

Thirty-five bacterial isolates were recovered from medical specimens of wound swabs from patients at Zagazig University Hospital (data not shown). Gram-negative and positive bacteria accounted for 30% and 70%, respectively. The resistance rates of the isolates to the tested antibiotics presented. They showed a low resistance to gentamycin (15%) followed by chloramphenicol (22%), and amikacin (25%). Otherwise, 85% and 80% of bacterial isolates were resistant to aztreonam and cephalexin, respectively. Among these isolates, *P. aeruginosa* and *S. aureus* were resistant to multiple antibiotics. *P. aeruginosa* was resistant to nitro-furantoin, doxycycline, vancomycin, aztreonam, cefotaxime, Imipenem, chloramphenicol, amoxicillin/clavulanic and trimethoprim-sulphamethoxazole. *S. aureus* was resistant to gentamycin, ceftazidime, ciprofloxacin, erythromycin, vancomycin, cephalexin, penicillin, and aztreonam. The most practical definition of MDR used for gram-negative and gram-positive bacteria is resistance to three or more antimicrobial classes [59]. MDR pathogens are now widespread in hospitals as well as in the environment and communities [60]. The World Health Organization (WHO), the Centers for Disease Control and Prevention (CDC), Infectious Diseases Society of America, and World Economic Forum have warned that antibiotic resistance is a global public health concern [61]. In recent times, a series of epidemics have made their mark in the antibiotic era generated by many resistant microorganisms such as penicillin-resistant *S. aureus*, methicillin-resistant *S. aureus* and vancomycin-intermediate *S. aureus*. The discovery and evolution of alternative therapeutic strategies against *P. aeruginosa* are increasingly sought and gaining more and more interest [62].

### Susceptibility of Multi-Drug Resistant Bacterial Isolate towards Metal NPs

The antimicrobial activity was assessed by zone of inhibition (ZOI) (Fig. 4). Analysis of variance of the effect of different concentrations of NPs was performed (Table S1 and S2). The CFF of *F. solani* KJ 623702 did not show any inhibitory effect. Gram-negative bacteria (*P. aeruginosa*) was more susceptible to AgNPs than gram-positive bacteria (*S. aureus*). The inhibition level was dose-dependent. The highest ZOI (22.4 mm) for *P. aeruginosa* *was* observed with 15 μg of AgNPs. ZnONPs showed the strongest antifungal activity against *F. oxysporum* (18.5 mm) (Fig. 7B). AgNPs and CuNPs exhibited a highly significant effect (*p* < 0.01) and a significant antibacterial effect (*p* < 0.05), respectively. Conversely, ZnONPs have a non-significant antibacterial effect (Table S1). All the synthesized NPs have a non-significant antifungal effect against the tested species (Table S2). Several studies reported that the synthesized NPs were utilized in the control of pathogenic micro-organisms. *P. aeruginosa* can cause disease in plants and animals, including humans. It is recognized for its ubiquity, intrinsically advanced antibiotic resistance mechanisms, and its association with serious illnesses. This study corroborates with [62-66] in which it was demonstrated that AgNPs have excellent antibacterial activity against *P. aeruginosa*. In general, the cell wall of gram-positive bacteria has a thick and rigid peptidoglycan layer (20-80 nm) and lipoteichoic acids which have a strong negative charge. For this reason, free Ag^+^ sequestrated, and thus fewer ions are able to reach the cytoplasmic membrane [67]. There are few studies on the antifungal activity of NPs against filamentous fungi. Padmavathy and Vijayaraphavan [68] proposed that the fungicidal activity of ZnONPs was due to destruction of cell membrane integrity by the abrasive surface of ZnONPs, which has defects such as edges and corners. Lipovsky *et al*. [69] supported the finding that ZnONPs provide a novel family of fungicidal compounds by creating singlet oxygen and hydroxyl radicals (ROS).

The MIC of AgNPs against *P. aeruginosa* was found to be 18.33 ± 2.89 (the most susceptible species) (Table 1). ZnONPs have the highest antifungal effect against *F. oxysporum* where the MIC was found to be 24.7 ± 2.80 μg/ml. Analysis of variance of the effect of different minimum inhibitory concentrations showed highly significant antimicrobial effect (Table S3).

### Transmission Electron Microscope

**Fig. 5:**
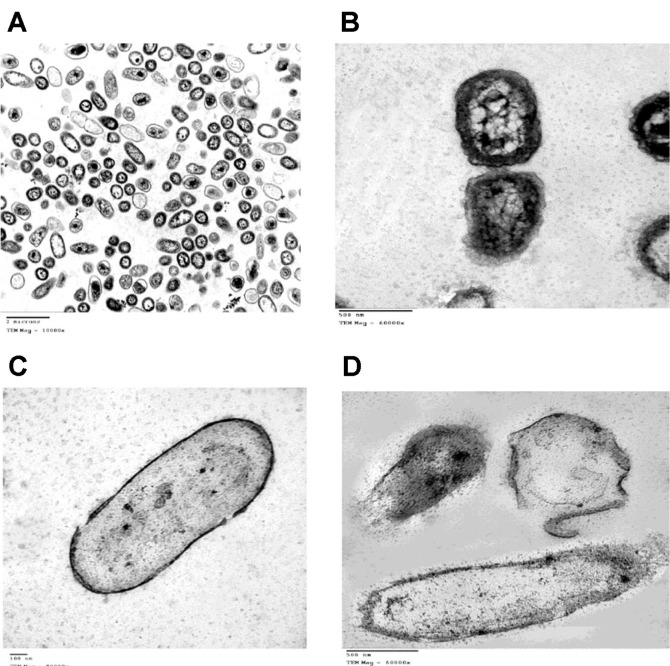
Transmission electron microscope (TEM) micrographs of *P. aeruginosa* control (**A**, **B**) and in response to AgNPs (**C**, **D**).

The electron micrographs of untreated and AgNP-treated *P. aeruginosa* cells are displayed in Fig. 5. TEM of untreated cells revealed a regular cell wall and uniform intracellular contents. Conversely, ultrathin sections of AgNP-treated cells revealed pits in the cell wall with the internalization of NPs (Fig. 5). Moreover, fragmentation, complete disappearance of cellular contents, disorganization and leakage of internal components were obvious. AgNPs exhibited antimicrobial potential through several mecha-nisms. They attach to the cell membrane and alter its structure, transport activity, penetrability, prompt neutralization of the surface electric charge and produce cracks and pits through which internal cell contents are effluxed [70]. Silver ions released by the NPs react with -SH of cell membrane components and bacterial enzymes to produce stable S-Ag bonds or disulfide bonds (R-S-S-R). Gopinath *et al*. [71] found that NPs can alter the signal transduction in bacteria by dephosphorylating the peptide substrate on tyrosine residues. Silver is a soft acid and the bacterial cells are made up of sulfur and phosphorus (soft bases). So, they tend to react with each other and interrupt DNA replication [72]. It was also reported that AgNPs bind to external proteins and hence create pores and form reactive oxygen species (ROS) [72].

### Pyocyanin Pigment

**Fig. 6:**
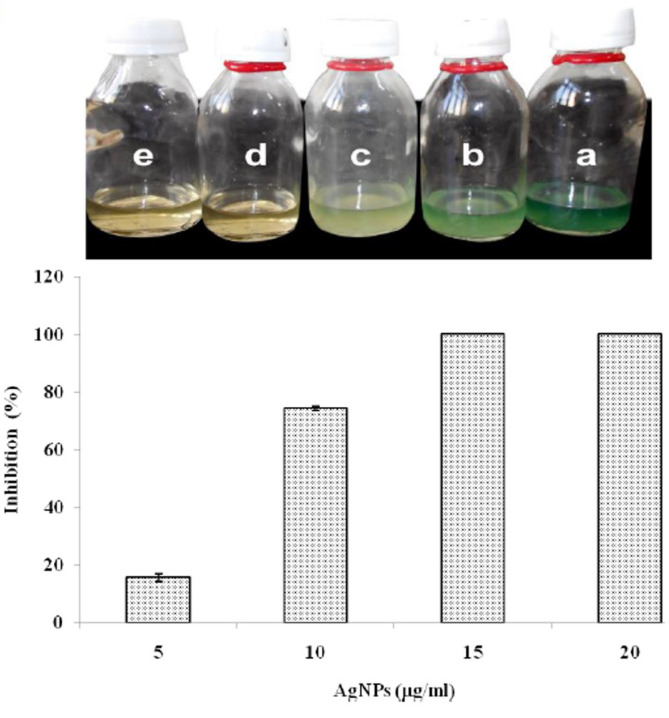
Biosynthesis of pyocyanin by *P. aeruginosa* in response to AgNPs (5, 10, 15, and 20 μg/ml). Visual inspection of broth culture of *P. aeruginosa* (upper panel), colorimetric concentrations of pyocyanin (lower panel) in response to AgNP concentrations.

This pigment is an important virulence factor produced by *P. aeruginosa* [73]. Production of pyocyanin was inhibited by AgNPs in a concentration-dependent manner (5-20 μg of AgNPs/ml) causing 15-100% suppression for pyocyanin (Fig. 6). Effect of different concentrations of synthesized AgNPs was highly significant (*p* < 0.001). Similar results were reported by Singh *et al*. [74].

In conclusion, *F. solani* KJ 623702 was used for the extracellular synthesis of AgNPs, CuNPs, and ZnONPs. Total cost-effectiveness and eco-friendly synthesis of nano-particles were reported. Ag^+^, Cu^++^, and Zn^++^ ions exposed to the CFF of *F. solani* were characterized by UV–visible spectrophotometer, TEM, FTIR, XRD, and Z-potential confirmed the reduction of ions to NPs. FTIR also demonstrated that protein might play a prominent role in the stabilization of NPs. The strong antimicrobial activity of the biogenic NPs against gram-negative bacteria (*E. coli*, *K. pneumonia*, and *P. aeruginosa*), gram-positive bacteria (*Enterococcus* sp. and *S. aureus*) and the filamentous fungi *A. awamori*, *A. fumigatus*, and *F. oxysporum* have been confirmed. AgNPs have a highly significant antibacterial effect against *P. aeruginosa*, while ZnONPs were the most effective against *F. oxysporum*. TEM micrographs of AgNP-treated *P. aeruginosa* showed cracks and pits in the cell wall, an internalization of NPs and liquefaction of cytoplasmic contents. Pyocyanin pigment produced by *P. aeruginosa* was entirely inhibited by 20 μg of AgNPs/ml.

## Supplemental Materials



Supplementary data for this paper are available on-line only at http://jmb.or.kr.

## Figures and Tables

**Table 1:** Minimum inhibition concentrations (MIC) of AgNPs, CuNPs and ZnONPs synthesized by *F. solani*.

Tested microbial species	MIC (μg/ml)			Significance

	AgNPs	CuNPs	ZnONPs	
*Pseudomonas aeruginosa*	18.33±2.89	41.67±5.77	28.3±2.52	.001
*Escherichia coli*	31.2±2.34	33.33±2.80	31.8±2.80	.729
*Klebsiella pneumoniae*	30±4.00	48.31±2.70	40±.00	.002
*Staphylococcus aureus*	46.7±2.75	31.67±2.25	21.7±2.12	.000
*Enterococcus sp*.	48.3±2.87	43.33±5.77	38.3±2.88	.002
*Aspergillus awamori*	40±.00	31.67±2.13	26.7±2.15	.001
*Aspergillus fumigatus*	43.3±2.13	41.60±3.21	28.3±3.60	.003
*Fusarium oxysporum*	35.2±4.22	40±.00	21.7±2.80	.001
