# Supplementary material for: Biocidal Activity of Metal Nanoparticles Synthesized by Fusarium solani against Multidrug-Resistant Bacteria and Mycotoxigenic Fungi
Source: J Microbiol Biotechnol. 2019 Aug 27;30(2):226–36. doi: 10.4014/jmb.1906.06070 (PMC9728364; doi:10.4014/jmb.1906.06070)
Supplement: Supplementary file 1 [file JMB-30-2-226-supple.pdf]

J. Microbiol. Biotechnol.
https://doi.org/10.4014/jmb.1906.06070

Table S1. Analysis of variance of the effect of different concentrations of biogenic AgNPs, CuNPs and ZnONPs synthesized by *F. solani* KJ 623702 on bacterial growth SS, sum of squares; df, degree of freedom; MS, mean square; Sig., Significance

Source	SS	df	MS	F	Sig.
Ag NPs					
Intercept	8691.670	1	8691.670	4788.506	.000
microorganism	192.688	4	48.172	26.539	.000
Groups	347.867	2	173.934	95.825	.000
microorganism * Groups	55.002	8	6.875	3.788	.004
Error	54.453	30	1.815		
Total	9341.680	45			
Cu NPs					
Intercept	3225.800	1	3225.800	2819.755	.000
microorganism	193.660	4	48.415	42.321	.000
Groups	145.456	2	72.728	63.573	.000
microorganism * Groups	20.384	8	2.548	2.227	.054
Error	34.320	30	1.144		
Total	3619.620	45			
ZnO NPs					
Intercept	5511.200	1	5511.200	5109.271	.000
microorganism	260.960	4	65.240	60.482	.000
Groups	230.476	2	115.238	106.834	.000
microorganism * Groups	14.824	8	1.853	1.718	.135
Error	32.360	30	1.079		
Total	6049.820	45			

Table S2. Analysis of variance of the effect of different concentrations of biogenic AgNPs, CuNPs and ZnONPs synthesized by *F. solani* KJ 623702 on fungal growth SS, sum of squares; df, degree of freedom; MS, mean square; Sig., Significance

Source	SS	df	MS	F	Sig.
Ag NPs					
Intercept	4004.053	1	4004.053	3665.969	.000
microorganism	52.607	2	26.303	24.082	.000
Groups	50.327	2	25.163	23.039	.000
microorganism * Groups	5.773	4	1.443	1.321	.300
Error	19.660	18	1.092		
Total	4132.420	27			
Cu NPs					
Intercept	1787.707	1	1787.707	4465.133	.000
microorganism	43.694	2	21.847	54.567	.000
Groups	32.547	2	16.274	40.647	.000
microorganism * Groups	.395	4	.099	.247	.908
Error	7.207	18	.400		
Total	1871.550	27			
ZnO NPs					
Intercept	5034.803	1	5034.803	4979.476	.000
microorganism	66.127	2	33.063	32.700	.000
Groups	82.247	2	41.123	40.671	.000
microorganism * Groups	5.413	4	1.353	1.338	.294
Error	18.200	18	1.011		
Total	5206.790	27			

Table S3. Analysis of variance of the effect of different minimum inhibitory concentrations had highly significant antimicrobial effect

Source	SS	Df	MS	F	Sig.
Between Groups	14225.582	3	4741.861	2534.625	.000
Within Groups	14.967	8	1.871		
Total	14240.549	11			

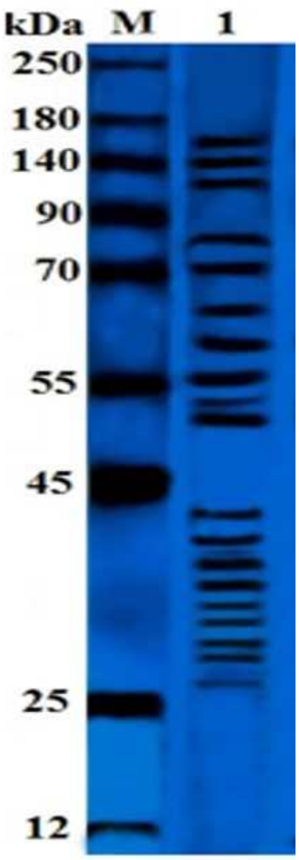

Fig. S1. SDS-PAGE profile of water extract of F. solani prior addition of salts of each compound.

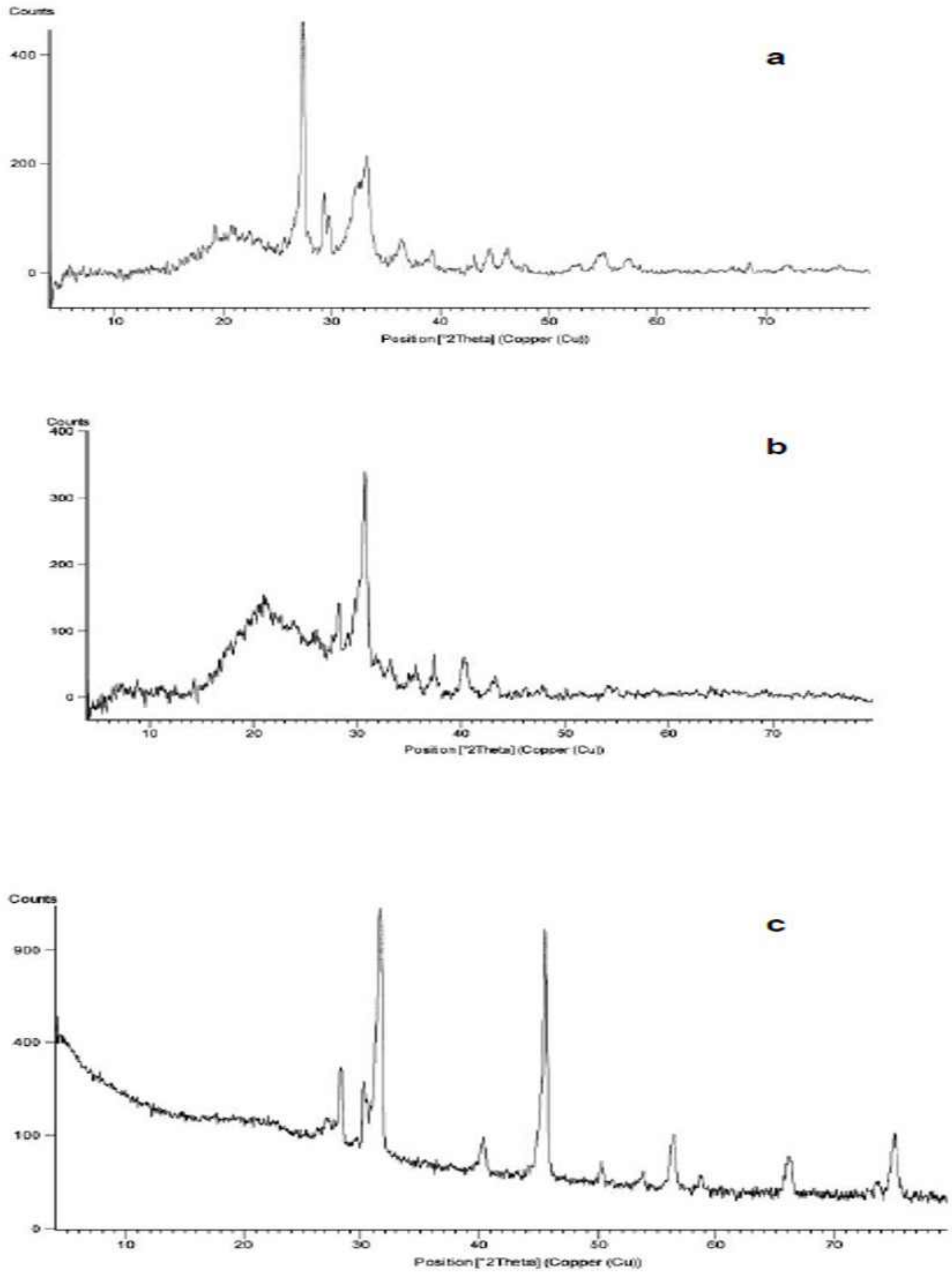

Fig. S2. Fourier infrared spectroscopy (FTIR) spectra of the cell-free filtrate of *F. solani* (A), AgNPs (B), CuNPs (C), and ZnONPs (D).

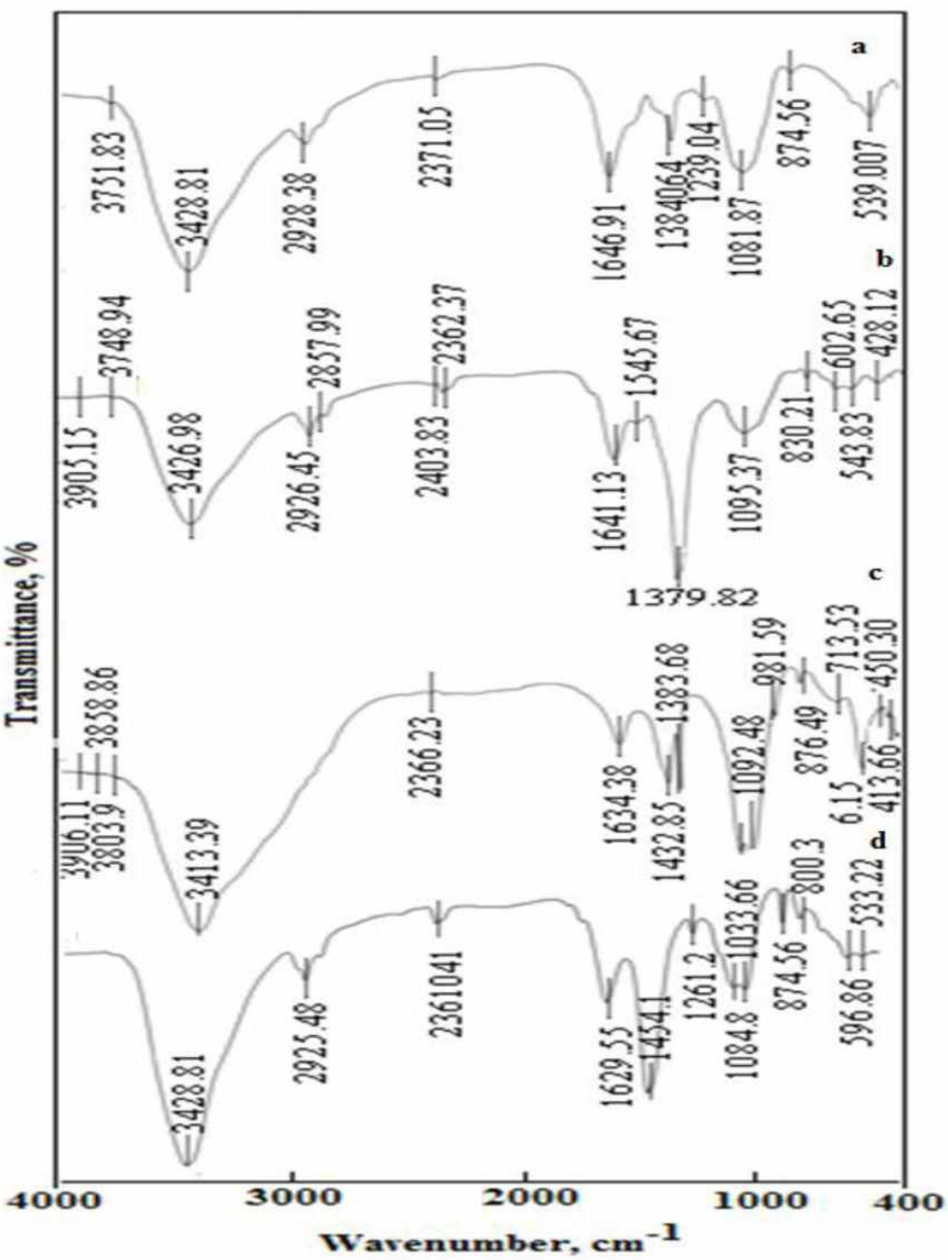

Fig. S3. Zeta potential analysis of AgNPs (A), CuNPs (B), and ZnONPs (C) synthesized by *F. solani.*
